# Indirect Genetic Effects and the Spread of Infectious Disease: Are We Capturing the Full Heritable Variation Underlying Disease Prevalence?

**DOI:** 10.1371/journal.pone.0039551

**Published:** 2012-06-29

**Authors:** Debby Lipschutz-Powell, John A. Woolliams, Piter Bijma, Andrea B. Doeschl-Wilson

**Affiliations:** 1 The Roslin Institute and Royal (Dick) School of Veterinary Studies, University of Edinburgh, Easter Bush, Midlothian, United Kingdom; 2 Animal Breeding and Genomics Centre, Wageningen University, Wageningen, Netherlands; Aarhus University, Denmark

## Abstract

Reducing disease prevalence through selection for host resistance offers a desirable alternative to chemical treatment. Selection for host resistance has proven difficult, however, due to low heritability estimates. These low estimates may be caused by a failure to capture all the relevant genetic variance in disease resistance, as genetic analysis currently is not taylored to estimate genetic variation in infectivity. Host infectivity is the propensity of transmitting infection upon contact with a susceptible individual, and can be regarded as an indirect effect to disease status. It may be caused by a combination of physiological and behavioural traits. Though genetic variation in infectivity is difficult to measure directly, Indirect Genetic Effect (IGE) models, also referred to as associative effects or social interaction models, allow the estimation of this variance from more readily available binary disease data (infected/non-infected). We therefore generated binary disease data from simulated populations with known amounts of variation in susceptibility and infectivity to test the adequacy of traditional and IGE models. Our results show that a conventional model fails to capture the genetic variation in infectivity inherent in populations with simulated infectivity. An IGE model, on the other hand, does capture some of the variation in infectivity. Comparison with expected genetic variance suggests that there is scope for further methodological improvement, and that potential responses to selection may be greater than values presented here. Nonetheless, selection using an index of estimated direct and indirect breeding values was shown to have a greater genetic selection differential and reduced future disease risk than traditional selection for resistance only. These findings suggest that if genetic variation in infectivity substantially contributes to disease transmission, then breeding designs which explicitly incorporate IGEs might help reduce disease prevalence.

## Introduction

Infectious diseases in livestock constitute a major threat to the sustainability of livestock production. Moreover, the need to contain epidemics has been further emphasized by the threat of transmission to other species – in particular humans – as illustrated in the recent swine flu epidemic [1]. Reducing disease prevalence through selection for host resistance offers a desirable alternative to chemical treatment which is a potential environmental concern due to run-off, and sometimes only offers limited protection due to pathogen resistance [2], [3]. However, control of infectious diseases through selection has proven difficult as genetic analyses of resistance to infectious disease from field data tend to report low heritabilities [4]. But is this a reflection of true genetic variance?

Current genetic analyses of disease data tend to focus on individual susceptibility to infectious disease, ignoring information from group members. However, using a stochastic epidemiological model, Nath *et al*. [5] identified the transmission rate, latent period and recovery period as critical parameters for the risk and severity of infectious disease. In other terms, Nath *et al*. [5] identified the impact that individuals have on each other as critical parameters for the risk and severity of infectious disease. Moreover, evolutionary theory would suggest that more genetic variation may be found in an individual's impact on its groupmates than in susceptibility. Since an individual's susceptibility is a component of its own fitness, natural selection works to exhaust heritable variation in susceptibility. An individual's impact on its groupmates, in contrast, is not a component of its fitness, and may therefore accumulate greater heritable variation [6]. As demonstrated by Van Dyken *et al.* [7] this would occur even when kin-selection is acting, as populations in kin selection-mutation balance contain a stable frequency of ‘cheaters’. In the context of disease, ‘cheaters’ correspond to hosts with increased shedding of infectious pathogens which has no damage to their own fitness but a potentially high cost to the herd. For example, assuming that animals with a greater parasite burden will also shed more, Raberg et al. [8] found genetic variation in anaemia and weight loss corresponding to increasing parasite burden of rodent malaria in laboratory mice. These arguments suggest that there is an opportunity in capturing genetic variation in host infectivity, which is the propensity of transmitting infection upon contact with a susceptible individual. Especially as, there is abundant evidence that heterogeneity in infectivity can profoundly impact upon disease prevalence in the population, with super-shedders being an extreme example [9]–[12].

Over the last forty years, the theory of Indirect Genetic Effects (IGE) has been developed to investigate the impact of interactions among individuals on the expression and evolution of traits [13]–[16]. An indirect genetic effect, also known as an associative or social genetic effect, is a heritable effect of an individual on the trait value of another individual [14]. Indeed, if an individual's trait value is affected by the genotypes of its population members (indirect genetic effect), then response to selection will be affected by these IGEs. It has been shown both theoretically [13], [14], [17] and experimentally [18] that IGEs can drastically affect the rate and direction of response to selection. In this context, host infectivity can be regarded as an indirect effect to disease status. Thus an individual's disease status and infectious disease prevalence in a population is likely to be affected by host genetic variation in both susceptibility and infectivity. To date, however, no work has been published examining the prospects of IGE models for infectious diseases, suggesting that part of the heritable variation underlying disease prevalence is overlooked.

Genetic variation in infectivity is difficult to measure directly and may need to be inferred from more readily available information such as binary disease data (infected/non-infected). Our hypothesis is that current genetic models applied to binary disease data do not capture the full genetic variation underlying disease prevalence and that a model including IGEs is more appropriate. This study, therefore, examines to what extent genetic variance in infectivity/susceptibility is captured by a conventional model versus an IGE model in populations with simulated genetic variation in infectivity, and whether selection on breeding values estimated with IGE models offer greater potential for reducing disease prevalence. In order to address this question, we modelled disease progression in populations with different genetic architectures for infectivity/susceptibility and estimated the genetic variance in the simulated binary disease data with a conventional animal model and a model including IGEs. Finally, we evaluated selection response in susceptibility and infectivity, and its impact on future disease risk, using the estimated breeding values (EBV) derived from both models.

## Methods

### The epidemiological model

An epidemic was simulated to describe disease progression in the population and provide as output the disease status of each individual at given time points. To avoid overburdening the results with unnecessary complexity we chose a simple compartmental stochastic SIR model of disease spread modified from [19]. In an SIR model it is assumed that individuals start as being susceptible (S) but may then become infected (I), upon contact with an infected individual, eventually recover (R) and are then no longer susceptible. The speed of transition between the epidemiological compartments S, I, R is determined by the transmission parameter β (S->I) and by the recovery rate γ (I->R). It was also assumed that infected individuals become immediately infectious. The contact between individuals within a group was constant and uniform (contact rate  = 1) and no transmission was allowed between groups.

To allow for individual genetic variation in the epidemiological parameters β and γ, each individual j was assigned its own level of susceptibility g_j_, infectivity f_j_ and speed of recovery 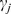. The pairwise transmission parameter β_jk_ was then defined as
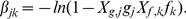
(1)


We refer to Text S1 for the derivation of equation (1). For ease of reading a comprehensive list of symbols and notation is given in Table 1. Thus *β_jk_* is a function of the product of the susceptibility *g* of individual *j* and the infectivity *f* of individual *k*. To reflect wether susceptibility is expressed by individual *j*, it is scaled by *X_g,j_* which equals one if *j* is susceptible and zero otherwise. Similarly, infectivity is scaled by *X_f,k_* which equals one if *k* is infected and zero otherwise. For simplicity, it was assumed that infectivity and susceptibility are independent, and that all individual speeds of recovery 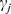 were assumed to be equal to a constant 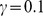 if the individual was infected and zero otherwise.

**Table 1:** Symbols and Notations.

g_j_	Susceptibility of individual j
f_j_	Infectivity of individual j
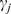	Speed of recovery of individual j
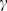	Speed of recovery constant
β_jk_	Pairwise transmission parameter from individual k to individual j
r_I_	Average rate of infection
r_R_	Average rate of recovery
X_1_,x_2_	Random variates
U(0,1)	Uniform distribution between zero and one
N	Population size
N	Group size
µ	Fixed mean of susceptibility and infectivity
G1	Effect of allele with small effect on susceptibility, in bi-allelic architecture
G2	Effect of allele with large effect on susceptibility, in bi-allelic architecture
F1	Effect of allele with small effect on infectivity, in bi-allelic architecture
F2	Effect of allele with large effect on infectivity, in bi-allelic architecture
MAF	Minor allele frequency (right-skewed distribution, applies to allele with large effect)
Α	Allele substitution effect
N(µ,σ^2^)	Normal distribution with mean µ and variance σ^2^
Г(a,θ)	Gamma distribution with shape a and scale θ
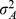	Genetic variance from conventional model
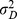	Direct genetic variance from IGE model
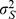	Indirect genetic variance from IGE model
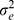	Residual variance
B_1_, b_2_	Regression coefficients
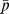	Mean number of infected groupmates
EBV_A_	Estimated Breeding Values from the conventional model
EBV_D_	Estimated Breeding Values for the direct effect from the IGE model
EBV_S_	EBV for the indirect effect from the IGE model
I_x_	Index of Estimated Breeding Values
R_0_	Basic reproduction number: expected number of secondary infections caused by an individual in its lifetime.

The epidemic was simulated as a Poisson process, i.e. as a series of random independent events occurring at given average rates in continuous time. In this model the possible events were infection of a susceptible individual and recovery of an infected individual. The average infection rate *r_I_* within a group was estimated as the sum of the pairwise transmission parameters *β_jk_* of the group members and the average recovery rate *r_R_* as the sum of the individual speeds of recovery 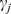.

The simulated epidemic was started by a single randomly chosen infected individual within each group of size *n* in an otherwise naïve population. The time to the next event (inter-event times) and the corresponding event type (infection of a susceptible individual or recovery of an infected individual) were then estimated using Gillespie's direct algorithm [20] which is a commonly used algorithm in stochastic epidemiological models [21]. Specifically, the inter-event times for each group were sampled from an exponential distribution with parameter 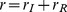. In other words, the time between each event was estimated as 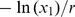 where *x_1_ ∼* U(0,1). The specific event type *v* (i.e. infection or recovery) which then occurs was obtained by drawing a random variate from a discrete distribution with probability 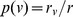. Hence, the event was an infection if 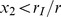 where *x_2_ ∼* U(0,1) and a recovery otherwise. The individual involved in each event was then chosen randomly weighted by the individuals' susceptibility or recovery rate. No transmission was assumed between groups.

### Simulated Populations

In order to ensure a high power to detect genetic variation, large populations with a relatively large family size and a family structure following e.g. dairy cattle were simulated. In particular, populations of size N = 100,000 were created with a paternal half-sib structure and no full sibs. All parents were assumed to be unrelated. The half sib family size was 100 individuals. Similarly, in order to ensure a high power to detect genetic variation, each population was divided into 10,000 groups of size 10 chosen at random without reference to pedigree.

Breeding values for susceptibility and infectivity were assigned to the individuals in the parental generation using different distributions to account for different underlying genetic architectures. For the first architecture it was assumed that genetic variation in susceptibility was controlled by a single bi-allelic locus and genetic variation in infectivity by another bi-allelic locus. Both loci were assumed to segregate independently. This architecture was used to encompass diseases affected by a major gene. For example, Houston *et al.* [22] found that a single quantitative trait locus (QTL) explained 98% of the additive genetic variation in susceptibility to infectious pancreatic necrosis (IPN) in Salmon. For the second architecture it was assumed that genetic variation in these traits is influenced by many alleles conferring a continuous distribution of effect sizes (possibly stemming from several loci).

Parametric statistical analyses usually assume normality. However, as shown by Lloyd-Smith et al. [11], the distribution of infectivity is often right-skewed. Moreover, skewed distributions allow for larger variation when the distribution is confined to positive values. Both types of genetic architectures were, therefore, considered with either a symmetrical or a right-skewed frequency distribution. In all four combinations (two alleles- symmetric, two alleles- skewed, multiple alleles – symmetric, multiple alleles – skewed) mean susceptibility and infectivity were fixed at *µ* = 0.22, as different population means would lead to different prevalence profiles. Fixing the means does however imply that populations with different genetic architectures have different input variances, and may thus not be directly comparable. However the focus of the study is comparison of animal models *vs*. IGE models within a genetic architecture.

#### Two alleles genetic architecture

For the bi-allelic architecture, it was assumed that the locus influencing susceptibility has two alleles each inferring a value of *G1* or *G2* and the locus influencing infectivity has two alleles each inferring a value of *F1* or *F2*. We also assumed additivity of allelic effects without dominance and that the population is in Hardy-Weinberg equilibrium. In other words, the genetic values for susceptibility (or infectivity) in the parental population were sampled from a discrete distribution with three possible values *G1+G1* (or *F1+F1*), *G1+G2* (or *F1+F2*) and *G2+G2* (or *F2+F2*). The shape of the distribution was defined through the minor allele frequency (MAF) which applied to the allele with a large effect (*F2, G2*). The values corresponding to each of the alleles were then chosen such that the population mean and the allele substitution effect *α* were kept constant. The same parameters were used for both infectivity and susceptibility to facilitate comparison of estimated genetic parameters. Table 2 shows the parameter values for the bi-allelic genetic architecture. The offspring’s’ breeding values were then generated by randomly allocating dams to sires and randomly choosing one allele from each parent.

**Table 2:** Parameters for Breeding Values Generation.

		M.A.F.	Allele values		α	Population mean *µ*	Variance
**Distribution**			*F1,G1*	*F2,G2*			
**Two alleles**	**Symmetric**	0.5	0.02	0.2	0.18	0.22	0.0162
	**Skewed**	0.2	0.074	0.254	0.18	0.22	0.0104
**Multiple alleles**	**Symmetric**	-	-	-	-	0.22	0.0049
	**Skewed**	-	-	-	-	0.22	0.0440

MAF applied to the alleles with a large effect (F2, G2).

#### Multiple alleles genetic architecture

For the multiple alleles architecture, it was assumed that there would be sufficient alleles contributing to the additive genetic values of susceptibility and infectivity in the parental population to be adequately approximated by a continuous probability density function.

For the symmetric frequency distribution, the breeding values for the parental population were sampled from the normal distribution N(*µ, σ^2^*). The parameter values were taken as µ = 0.22 (i.e. the same as for the bi-allelic architecture) and *σ^2^* = 0.005 to avoid frequent negative values of susceptibility/infectivity (Table 2). If a negative value was sampled, it was discarded and re-sampled. Each offspring was allocated a breeding value equal to the mean of its parents plus a Mendelian sampling term.

For the skewed frequency distribution, the breeding values of the parental population for susceptibility and infectivity were assumed to be distributed according to the gamma distribution Г(*a,θ*). It is not possible to represent Mendelian inheritance by adding a Mendelian sampling term with a gamma distribution, however, as the offspring generation would no longer follow the same distribution as the parental generation. It was therefore assumed that the parental breeding values stem from ten additive loci with a large number of alleles each, whose values follow the gamma distribution Г(*a/20,θ*). The offspring were then randomly assigned one allele from each parent for each locus. The breeding values of the offspring are therefore distributed following Г(*a,θ*). Specifically, the parameters were taken as *a* = 1.1 and *θ* = 0.2 such that the mean *aθ* = *µ* = 0.22, i.e. the same as for the bi-allelic architecture, the variance *aθ^2^* = 0.044 (Table 2) and the distribution is right-skewed (skewness 2/*√a = *1.9).

For all populations, it was assumed that susceptibility and infectivity are fully heritable and that the outcome, i.e. whether an individual becomes infected or not, depends on both, the genetics and environment. The environmental contribution to the phenotypic variance was represented through the stochastic events (infection, recovery) in the epidemiological model. Thus, the model assumes genetic predisposition whilst maintaining full environmental stochasticity of the epidemics. Moreover, adding additional environmental noise would not provide further useful information to this study and would make it harder to interpret the results. Each architecture was run with variation introduced in susceptibility only, infectivity only, both or neither. When no variation in susceptibility/infectivity was introduced, all individuals were given a fixed breeding value of *µ* = 0.22 for that underlying trait. As each simulated population is divided into 10,000 groups, i.e. 10,000 independent epidemics, each simulation was replicated ten times.

#### Estimating genetic variance

Genetic variation between individuals was estimated from binary records which were obtained by recording the disease state of simulated individuals. The binary disease trait, disease presence, was one if an individual had become infected prior to a considered time-point and zero otherwise. The data were analysed at the same timepoint for all groups, which was the time at which 50% of individuals would have become infected in a homogeneous population with the same mean values for the input parameters. All analyses were carried out using ASRem [23].

To reflect current practise, genetic variance in disease presence was first estimated with a mixed model including a single genetic variance. In order to be in line with the indirect genetic effect model, this was achieved with an animal model for disease presence *y* observed in offspring *j* of sire *i*,
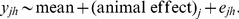
(2)


The group effect is absorbed by allowing for a correlation between the residuals of group members, this is equivalent to fitting a random group effect [24]. The animal variance is denoted as *σ_A_^2^*. Hereafter this model is referred to as the conventional model.

To estimate the genetic variance in the indirect effect, the data were analysed using the model developed by Muir *et al*. [16]. Thus for disease presence *y* observed in offspring *j* with this individual living in group *h* of size *n* with groupmates m,
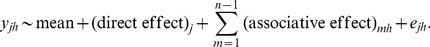
(3)


Similarly to the conventional model, the group effect is absorbed by allowing for a correlation between the residuals of group members [24]. Note that this model does not take account of the disease status of individuals *j* and their group members *m*, in other words, it is assumed that all individuals express the direct effect (susceptibility) and the associative effect (infectivity) at all times, regardless of their infection status. The variance of the direct and indirect genetic effects are denoted *σ_D_^2^* and *σ_S_^2^* respectively. Hereafter this model is referred to as the Indirect Genetic Effects (IGE) model.

#### Association between variation in susceptibility/infectivity and variation in binary disease presence

In order to assess to what extent the available genetic variation is being captured by the different statistical models, an estimate of expected output variance as a function of the input variance in infectivity/susceptibility is required. Following Dempster and Lerner [25] a linear relationship was assumed between input and output traits to provide an approximation. In particular, it was assumed that there is a linear relationship between disease presence in an individual *j* and that individual's susceptibility *g_j_* and the sum of the infectivities *f* of the *p* infected groupmates of that individual,
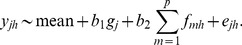
(4)


The regression mean and coefficients *b_1_* and *b_2_*, were estimated using this linear model in the statistical package R [26] with the known input (i.e. true *f* and *g* values) and output (*y*) data from the simulations. Hence the model in Equation 4 was used to estimate the true linear effects of infectivity and susceptibility to the observed binary disease presence.

The number of groupmates that have been infected (p) is a variable which depends on the group *h* and status of individual *j*. Indeed, if in a given group x individuals have been infected, individual *j* will have *x* groupmates which have been infected if it is susceptible and *x−1* if it is one of the infected individuals. The variance of disease presence *σ^2^* may therefore be expressed as follows (cf. derivation in Text S2):
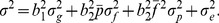
(5)


This expression can be compared with the estimated variance of disease presence 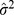 that is obtained from the IGE model in equation (3),
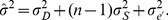
(6)


The first term in equation (5) is a function of the input variance in susceptibility 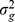 and should be approximately comparable to 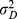 from the IGE model and to 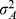 from the conventional model. The second term in equation (5) is a function of the input variance in infectivity 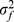 and mean number of infected groupmates 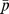 over all groups, and should be approximately comparable to 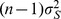, i.e. the second term in equation (6). The third term is a function of the squared input mean infectivity 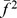 and the variance in number of infected groupmates 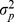 It is not directly comparable with any ASReml output as this term includes both between group variation and interaction between infectivity and susceptibility. Note that the expression of infectivity depends on the individual being infected, which in turn depends on the individuals' own susceptibility, and 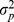 can be said to be the variation in numbers of individuals expressing infectivity. The interdependence in this model between infectivity and susceptibility is likely to be partly captured through a non-zero covariance estimate between direct and indirect genetic effects in ASReml [23].

### Estimated response to selection

In order to estimate response to selection based on Estimated Breeding Values (EBVs) derived from the conventional and IGE models, the impact of selection on true mean susceptibility/infectivity was examined. Here the population mean susceptibility/infectivity was compared to the mean susceptibility/infectivity after selection of 10% of the individuals with the lowest EBVs obtained from each model. For the conventional model, selection used the only available EBV (EBV_A_). For the IGE model, selection was based on the EBVs for direct (EBV_D_) and indirect (EBV_S_) genetic effect separately as well as for the index I_x_  =  EBV_D_ + (n−1) 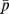 EBV_S_. The weight of the index was selected to take the mean level of exposure i.e. (n−1) 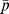 into account.

To quantify response to selection in terms of risk and severity of the epidemic, the basic reproduction number R_0_ was estimated for the whole population and for each selected subpopulation using the true values of susceptibility and infectivity. R_0_ is the mean number of secondary infections an infected individual will cause in its lifetime and is commonly used as a measure of disease risk and severity in epidemiology [27]. By definition, an epidemic will die out if R_0_<1. Following a SIR model for a closed population, 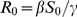, with *S_0_ = (n−1)* being the initial number of susceptible individuals in a group [19]. Incorporating equation (1) and taking a Taylor series expansion we obtain,
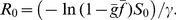
(7)


The symmetry of susceptibility and infectivity in equation (7) implies that a decrease in mean susceptibility or infectivity will decrease mean R_0_ equally (cf. Figure S1, Text S3).

## Results

### Estimated genetic variance in disease presence using a conventional model

The estimated variances in disease presence obtained for each population using a conventional model, along with the mean presence over all groups in all replicates, are displayed in Table 3. Overall the variance estimates depend on input variance and on mean presence at time of evaluation. As input parameters were the same for susceptibility and infectivity, variance estimates along the rows of Table 3, where mean presence is the same, are directly comparable. Note that values in rows are not directly comparable, on the other hand, across columns with different mean presence.

**Table 3:** Estimated Genetic Variance in Disease Presence (Binary) Using a Conventional Animal Model.

			Variation introduced in:			
Distribution			None	Infectivity	Susceptibility	Both
**Two alleles**	**Symmetric**	Variance	0.32#±0.08	0.63#±0.09	25.35±0.27	18.74±0.45
		Mean presence	0.56	0.50	0.51	0.46
	**Skewed**	Variance	0.32#±0.08	0.37#±0.10	8.28±0.14	7.96±0.13
		Mean presence	0.56	0.53	0.53	0.51
**Multiple alleles**	**Symmetric**	Variance	0.13#±0.04	0.09#±0.08	0.12#±0.09	0.10#±0.03
		Mean presence	0.51	0.48	0.49	0.50
	**Skewed**	Variance	0.24#±0.08	0.74±0.09	31.02±0.53	18.56±0.45
		Mean presence	0.51	0.42	0.42	0.35

All parameters as in table 2. 10000 groups of size 10, ‘#’ means not significantly different from zero (P>0.05), values scaled by 10^3^.

The results indicate that, if there is variation in infectivity only, the conventional model fails to pick up the heritable variation in binary disease presence present in the data. Only in the populations with a skewed multiple allele genetic architecture (i.e. large variance in infectivity) a small amount of genetic variation is captured when there is variation in infectivity only. However, the resulting variance estimate was only 3.5% of that compared to populations with the same variance introduced in susceptibility.

### Estimated genetic variances using an IGE model

Given that the prevalence profiles were similar between genetic architectures (cf. Figures S2 & S3, Text S3) and the skewed multiple alleles population had the largest input variance, analyses using the IGE model were only performed on populations with skewed distributions for susceptibility and infectivity.

The genetic parameters obtained by analysis with the IGE model along with relevant statistics are displayed in Tables 4 & 5. Note that following equation (6) the contribution of the indirect genetic effect to the phenotypic variance is (n−1) times greater than the values in Tables 4 & 5. Variance in infectivity is captured by the *σ_S_^2^*, in populations with both genetic architectures (cf. Tables 4 & 5). A log-likelihood test was performed to evaluate the statistical significance of the indirect genetic effects term. As would be expected, the indirect genetic effects term was significant (P<0.05) in populations with variation in infectivity (cf. Table 5). The analysis of the skewed multiple alleles population also implies that there is a statistically significant positive genetic covariance between the direct and the indirect effect when there is variance in susceptibility (cf. Tables 4 & 5), despite susceptibility and infectivity being independent in our simulation. This is probably due to the fact that the model fitted assumes constant expression of effects by all group members whereas an individual will only express infectivity if infected, which will depend on the individual's susceptibility. Note that the values in Tables 4 & 5 were obtained from the same data as those in Table 3, so the values in Table 3 can be compared to those in Tables 4 & 5.

**Table 4:** Estimated Genetic Variance in Disease Presence (Binary), in Populations with a Skewed Bi-Allelic Genetic Architecture Underlying Susceptibility/Infectivity, Using the Indirect Genetic Effects Model.

	Variation introduced in:			
Estimated genetic variance/covariance in:	None	Infectivity	Susceptibility	Both
**Direct effect** 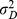	0.32#±0.09	0.22#±0.11	9.19±0.30	8.63±0.16
**Indirect effect** 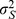	0.14#±0.04	0.51±0.04	0.16#±0.03	0.43±0.05
**Direct/indirect effect** 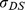	0.06#±0.04	0.08#±0.08	0.45#±0.14	0.59#±0.13
Log likelihood test P-value	0.4	0.3*10^−2^	0.5	0.04
Mean presence	0.56	0.53	0.53	0.51

Values scaled by 10^3^, ‘#’ means not significantly different from zero (P>0.05). Values along the rows are directly comparable to each other where mean presence is the same. Estimates averaged over ten iterations. Parameter values as in Table 2, 10000 groups of size 10. The log-likelihood P-value refers to the significance of the indirect genetic effect.

**Table 5:** Estimated Genetic Variance in Disease Presence (Binary), in Populations with a Skewed Multiple Alleles Genetic Architecture Underlying Susceptibility/Infectivity, Using the Indirect Genetic Effects Model.

	Variation introduced in:			
Estimated genetic variance/covariance in:	None	Infectivity	Susceptibility	Both
**Direct effect** 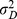	0.26#±0.09	0.36#±0.11	28.07±1.97	19.55±0.47
**Indirect effect** 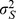	0.16#±0.03	1.00±0.09	0.11#±0.04	0.43±0.04
**Direct and indirect effect** 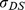	0.08#±0.05	0.13#±0.09	1.05±0.29	0.86±0.09
Log likelihood test P-value	0.5	0.2*10^−5^	0.5	0.3*10^−2^
Mean presence	0.51	0.42	0.42	0.35

Values scaled by10^3^, ‘#’ means not significantly different from zero (P>0.05). Values along the rows are directly comparable to each other where mean presence is the same. Estimates averaged over ten replicates. Parameters as in Table 2, 10000 groups of size 10. The log-likelihood P-value refers to the significance of the indirect genetic effect.

### Comparison of input and estimated variances

Input variance in susceptibility and infectivity and estimated variances were brought to a comparable scale using equations (5) and (6) and are displayed in Table 6. From Table 6 it is evident that the first term in equation (5), *σ_D_^2^*, and *σ_A_^2^* are approximately similar. However, the second term in equation (5) appears to be consistently larger than (n−1)*σ_S_^2^*, suggesting that the IGE model underestimates variation in infectivity. This could be due to the fact that the IGE model assumes constant expression of infectivity by all group-members, whereas in equation (5) expression of infectivity is limited to infected individuals. In this way the indirect effect is distributed between (*n*−1) individuals, in the genetic analysis with the IGE model, compared to 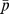 in equation (5) with 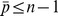. The discrepancy in these variance estimates suggests that there is some scope for improvement.

**Table 6:** A Comparison of Expected and Observed Variance Components for the Skewed ‘Multiple Alleles’ and ‘Two Alleles’ Architectures When Genetic Variance Is Introduced INTO Infectivity, or Susceptibility, or Both.

		Expected:	IGE:	Conventional:	Expected:	IGE:
Variation introduced in:		Susceptibility	Direct		Infectivity	Indirect
		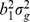	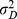	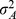	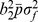	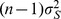
Multiple Alleles	Infectivity	0.00	0.36^#^	0.74	15.46	9.04
	Susceptibility	36.46	28.07	31.02	0.00	0.99^#^
	Both	20.39	19.55	18.56	9.20	3.87
Two Alleles	Infectivity	0.00	0.22^#^	0.37^#^	6.34	4.59
	Susceptibility	8.86	9.19	8.60	0.00	1.44^#^
	Both	7.92	8.63	7.96	5.34	3.87

Observed components are taken from results of analyses of data with either a conventional model (Eqn 2) or IGE model (Eqn 3), whilst expected components are obtained from the true simulated values and Eqn 5. ‘#’ means not significantly different from zero (P>0.05), values scaled by 10^3^.

### Impact of selection on mean susceptibility/infectivity and future disease risk

Mean susceptibility and infectivity, of the whole population and selected sub-populations, together with their respective average R_0_ values are displayed in Table 7. In line with our previous results, selection on the breeding values derived from the conventional model or on EBV_D_ alone, only reduces mean susceptibility (cf. Table 7). Less predictably, however, selection on EBV_S_ reduced both mean infectivity and susceptibility (cf. Table 7). This may be due to expression of infectivity being dependent on being infected, which in turn depends on susceptibility as mentioned above. This suggests that, when status isn't taken into account, selection targeting infectivity would indirectly also select for lower susceptibility. However, the resulting average R_0_ values displayed in Table 7 suggest that an index with both direct and indirect breeding values would create the greatest impact for the reduction of disease in future generation.

**Table 7:** Mean Susceptibility and Infectivity following Selection Using the Conventional Animal Model or the Indirect Genetic Effects Model.

Selection			Mean susceptibility	Mean infectivity	R_0_
**None**			0.22	0.22	4.46
**Conventional animal effect**		EBV	0.10	0.22	1.99±0.04
**Direct effect**		EBV_D_	0.10	0.22	1.96±0.04
**Indirect effect**		EBV_s_	0.15	0.17	2.38±0.11
**Index**	Ix = EBV_D_+ 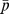 (n−1) EBV_s_		0.11	0.19	1.91±0.03

Population with variation in both infectivity and susceptibility following a skewed multiple allele genetic architecture. 10000 groups of size 10. Proportion selected was 0.10. Values ± standard error when greater than 0.005.

## Discussion

The hypothesis of this study was that low heritability estimates of disease traits may not reflect the true additive genetic variation inherent in a population, but rather a deficiency in the philosophy underpinning the models that are currently fitted. The aim of this study was therefore to assess whether it is possible to capture genetic variation in infectivity, when it is inherent in the data, with current statistical methods (animal/sire and IGE model). This was assessed for a variety of genetic architectures underlying susceptibility and infectivity. Our results show that, unlike a conventional model, which does not capture the variation in infectivity when it is presesnt in the data, a model which takes indirect genetic effects (IGE) into account captures some, though not all, of the inherent genetic variation in infectivity. This implies that, failing to include indirect genetic effects when analysing disease data from field studies may result in substantial genetic variation being missed. For example had the QTL, explaining 98% of the additive genetic variation in susceptibility to pancreatic necrosis in Salmon, found by Houston et al. [22] affected infectivity rather than susceptibility it would probably have been overlooked. Moreover, this additional genetic variance does not come at the expense of obtaining reliable estimates for genetic variance in susceptibility.

Our results show further that the ability of IGE models to detect genetic variance in infectivity can impact on response to subsequent artificial selection. From the mean susceptibility/infectivity and R_0_ values of the selected subsets of the population it is evident, that even with BVs estimated with the current IGE model based on binary data from a single time point, a greater impact on disease risk and severity could be achieved than by using BVs estimated with a conventional model. This is particularly true in populations with variation in infectivity only, as no selection would have been possible based on breeding values derived from a conventional model. At present, it is unknown whether infectivity harbours substantial genetic variation, or whether populations with genetic variation in infectivity only are common. This work, however, provides the first tools to address these questions.

Comparison with expected genetic variance from an alternative model using linear approximations suggests that there is still scope for improvement in applying IGE models to disease data. The apparent underestimation of genetic variance in infectivity may be due to the fact that the current methodology does not allow for status dependence. This could potentially cause an underestimation of the variance in infectivity as the indirect genetic effect is attributed to all individuals in a group when in reality it will have been expressed by only a subset of group members. Furthermore, our analysis revealed that the statistical model applied here is likely to yield a positive covariance estimate despite susceptibility and infectivity being independent. This is probably because expression of infectivity is state dependent and thus partly depends on the individual's susceptibility. Allowing for status dependency should therefore improve the accuracy of the estimated genetic parameters, suggesting that responses to selection may be greater than values presented here when methods are further improved.

The data of this study were generated using a standard epidemiological SIR model, assuming only host genetic variation in susceptibility and infectivity and full independence and heritability for both traits, in order to reduce unnecessary noise. Moreover, potential host-pathogen interactions were not considered. Although these assumptions may be representative for a variety of infectious diseases and populations, one would expect that the different sources of variances for diseases with more complex epidemiological patterns and in populations with more complex variance and co-variance structure would be more difficult to capture. This enhances the need for further investigations of IGE models with regards to requirements for data collection and experimental design for obtaining reliable genetic parameter estimates corresponding to host susceptibility and infectivity.

In addition to susceptibility and infectivity investigated here, there may be other sources of host genetic variation contributing to genetic variance of disease data and thus amenable for selection. For example, in addition to variation in infectivity, i.e. the propensity of individuals to infect others upon contact, genetic differences in transmission patterns may be caused by heritable variation in contact rate due to behavioural traits such as aggression or promiscuity. Previous studies have demonstrated [24] that IGE models are able to provide reliable estimates for these social interactions. Moreover, additional heritable variation in disease presence may come from genetic differences in recovery time among individuals, which affects their infective period. Analyses accounting for genetic differences in the length of the infective period may contribute to achieving greater response to selection, and emphasizes the scope for additional work in this area. We achieved a first step in understanding and extending the range of epidemiological parameters under potential host genetic influence that can be estimated with current quantitative genetic models. Further work is required to increase our understanding and improve the statistical models through the use of simulations and the application to field data.

Bishop and Woolliams [4] have shown that accuracy of genetic parameters for disease data obtained from field studies depends largely on exposure, and thus on time of measurement. Disease records obtained at a time corresponding to high disease prevalence are expected to give higher heritability estimates than disease records obtained at times when prevalence was low. It is expected that similar relationships also apply for the estimation of genetic parameters associated with indirect genetic effects. Further, Bijma [28] has shown that substantial improvement in accuracy of indirect genetic variance components can be achieved by optimising group size and composition. Since group size has a strong effect on disease progression between individuals and thus on prevalence patterns (cf. Text S3, Figures S2 & S3), it is expected that much improvement in the estimation of indirect genetic effects could be obtained by choosing the correct combination of group size and time at which records are collected. This could be combined with groups composed of members of two families, which yields much better accuracy of estimated genetic parameters than groups composed at random, particularly when groups are large [28]. Moreover, different weightings for the direct and indirect effects EBVs in the index might offer further improvements depending on the context.

One of the remaining challenges of analysing binary disease data with an IGE model is to establish the relationship between underlying susceptibility/infectivity and direct/indirect genetic effects. There are two standard ways of estimating genetic parameters from a binary trait, either using a linear mixed model, which treats the data as continuous and includes random factors, or a generalised linear model (GLM). The use of a GLM in combination with random factors (GLMM) is an area that is open to question. In fact, ASReml [23], the software used to fit the models in this paper, provides a warning not to use a GLM in combination with random factors. The relationship between the underlying traits, susceptibility and infectivity, and the observed trait disease presence is complex and stochastic. It is therefore questionable whether canonical link functions relating underlying parameters (e.g. susceptibility, infectivity) with the probability of observing an event (e.g. becoming infected), such as probit or logistic functions, would be appropriate in our case. In fact variance estimates obtained using a logistic model are not on the same scale as susceptibility and infectivity (cf. Text S4, Table S2). Moreover, should we use non-standard distributions and link functions, further statistical issues would arise, e.g. decomposing the phenotypic variance into genetic and environmental components may no longer be valid. Hence there is no theoretical apparent benefit in applying specific link functions with a GLM. Moreover, variance estimates obtained with a logistic model are qualitatively the same as those obtained with the linear models (cf. Text S4, Table S2). Besides, selection on the EBVs obtained with a logistic model provided no better results with regards to R_0_ (cf. Text S4, Table S3). We therefore decided to use a linear mixed model, which have been shown to provide estimates of genetic parameters of sufficient accuracy to generate selection response (e.g. [29], [30]).

Better understanding of the factors involved in indirect genetic effects to disease presence could open up further potential for disease control through selection. For example, it has been shown that, when indirect genetic effects occur, response to selection depends on the covariance between the direct and indirect genetic effects [14], which correspond to susceptibility and infectivity in our study. In this study we assumed that infectivity and susceptibility are independent. However, should they be positively correlated, the expected response to selection would be greater than indicated here. Doeschl-Wilson *et al*. [31] demonstrate for gastro-intestinal parasitism in sheep that correlation between underlying disease traits can have profound impact on heritabilities of observable disease traits and thus on response to selection. Moreover, a recent study showed molecular evidence for a positive correlation between susceptibility and infectivity as the known immunosuppressant stress hormone norepinephrine was shown to cause increase shedding of Salmonella [32]. It is therefore reasonable to believe that being able to estimate variance in indirect genetic effects for disease may open up new avenues for the control of infectious diseases through selection. In conclusion, this is the first work on the relevance of indirect genetic effects for the spread of infectious disease and it indicates that their relevance extends beyond behavioural interactions among individuals, which is the current focus of such research (e.g. [33]).

## Supporting Information

Figure S1 **Predicted disease prevalence over time.** Homogeneous population for suceptibility (high g  = 0.4, low g  = 0.04) and infectivity (high f  = 0.4, low f  = 0.04). Population consists of 500 groups of size 40 as in Table S1. Prevalence was averaged over all groups over three iterations. Probability of disease emerging in a group was 0.38 in the population with low susceptibility and infectivity and 1 for the other populations. The expected course of the epidemic is identical for high infectivity/low susceptibility and low infectivity/high susceptibility.
(TIF)

Figure S2 **Disease prevalence over time assuming many underlying alleles of varying effect coding for susceptibility or infectivity and a skewed distribution.** Parameters as in Table 2. Population structure parameters as in Table S1. A) groupsize of 10 B) groupsize of 40 C) groupsize of 400.
(TIF)

Figure S3 **Disease prevalence over time assuming two alleles code for susceptibility or infectivity and a symmetrical distribution.** Parameters as in Table 2. Population structure parameters as in Table S1. A) groupsize of 10 B) groupsize of 40 C) groupsize of 400.
(TIF)

Table S1 **Population structure parameters.**
(DOCX)

Table S2 **Variance estimates using a logistic link function.**
(DOCX)

Table S3 **Mean susceptibility and infectivity following selection using the conventional animal model or the Indirect Genetic Effects model with a logistic link function.**
(DOCX)

Text S1 **Derivation of transmission parameter from first principles.**
(DOC)

Text S2 **Derivation of variance in disease presence.**
(DOC)

Text S3 **Impact of model parameters on prevalence profiles.**
(DOC)

Text S4 **Impact of a logistic regression on variance estimates and selection response.**
(DOC)
