# Supplementary figures and images for: Indirect Genetic Effects and the Spread of Infectious Disease: Are We Capturing the Full Heritable Variation Underlying Disease Prevalence?
Source: PLoS One. 2012 Jun 29;7(6):e39551. doi: 10.1371/journal.pone.0039551 (PMC3387195; doi:10.1371/journal.pone.0039551)

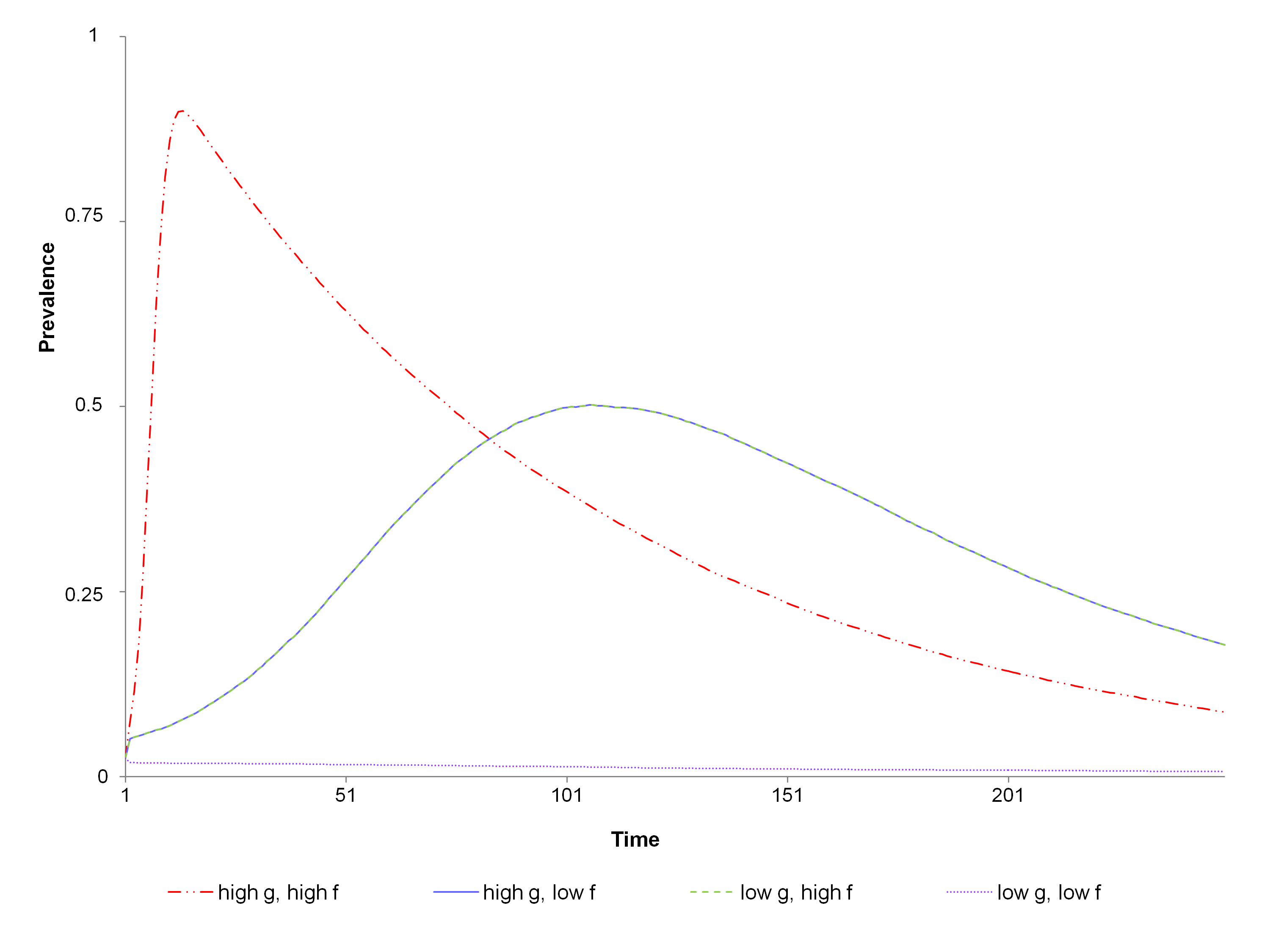

Supplement: Figure S1 — Predicted disease prevalence over time. Homogeneous population for suceptibility (high g = 0.4, low g = 0.04) and infectivity (high f = 0.4, low f = 0.04). Population consists of 500 groups of size 40 as in Table S1. Prevalence was averaged over all groups over three iterations. Probability of disease emerging in a group was 0.38 in the population with low susceptibility and infectivity and 1 for the other populations. The expected course of the epidemic is identical for high infectivity/low susceptibility and low infectivity/high susceptibility. (TIF) [file pone.0039551.s001.tif]

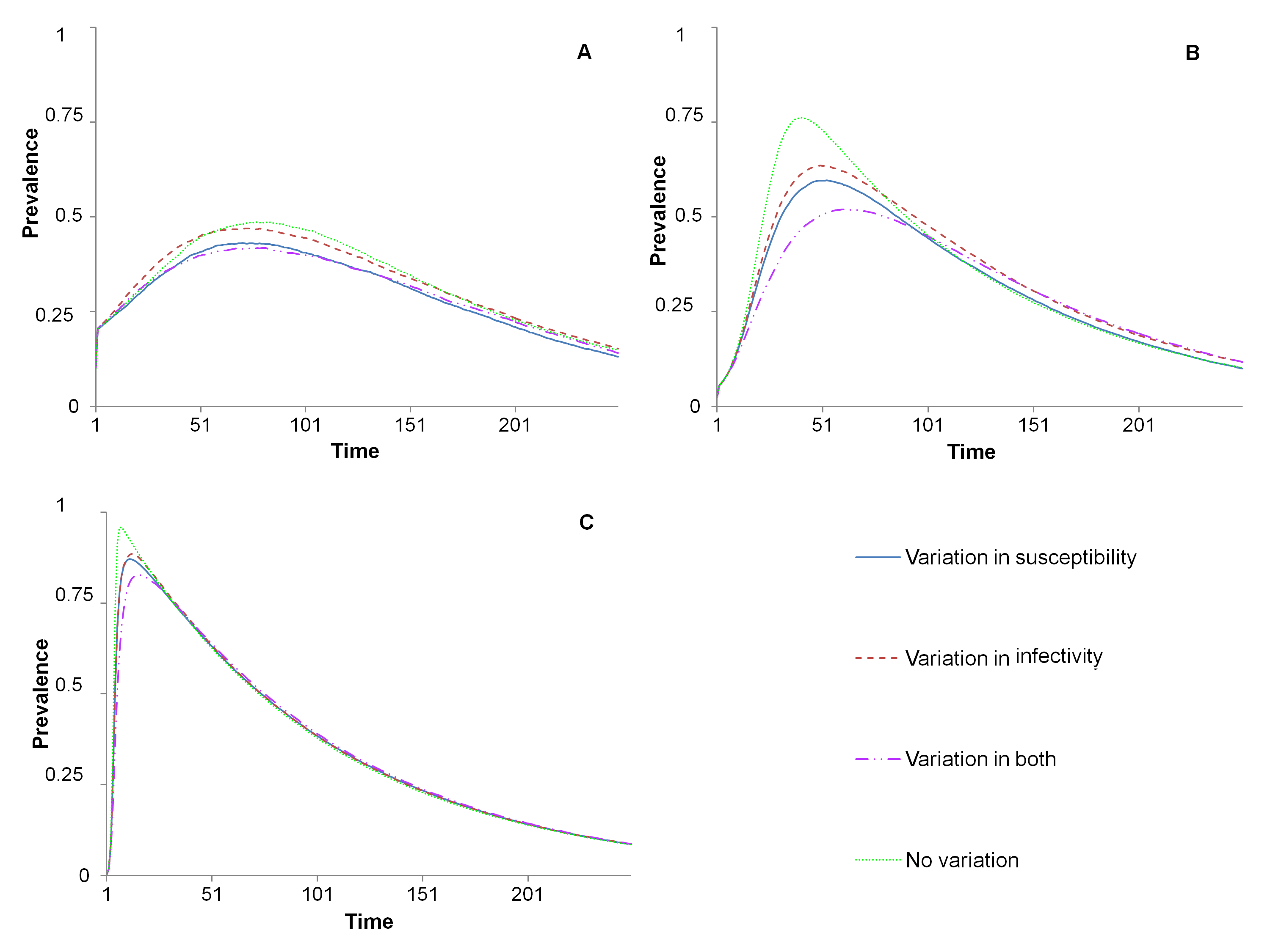

Supplement: Figure S2 — Disease prevalence over time assuming many underlying alleles of varying effect coding for susceptibility or infectivity and a skewed distribution. Parameters as in Table 2. Population structure parameters as in Table S1. A) groupsize of 10 B) groupsize of 40 C) groupsize of 400. (TIF) [file pone.0039551.s002.tif]

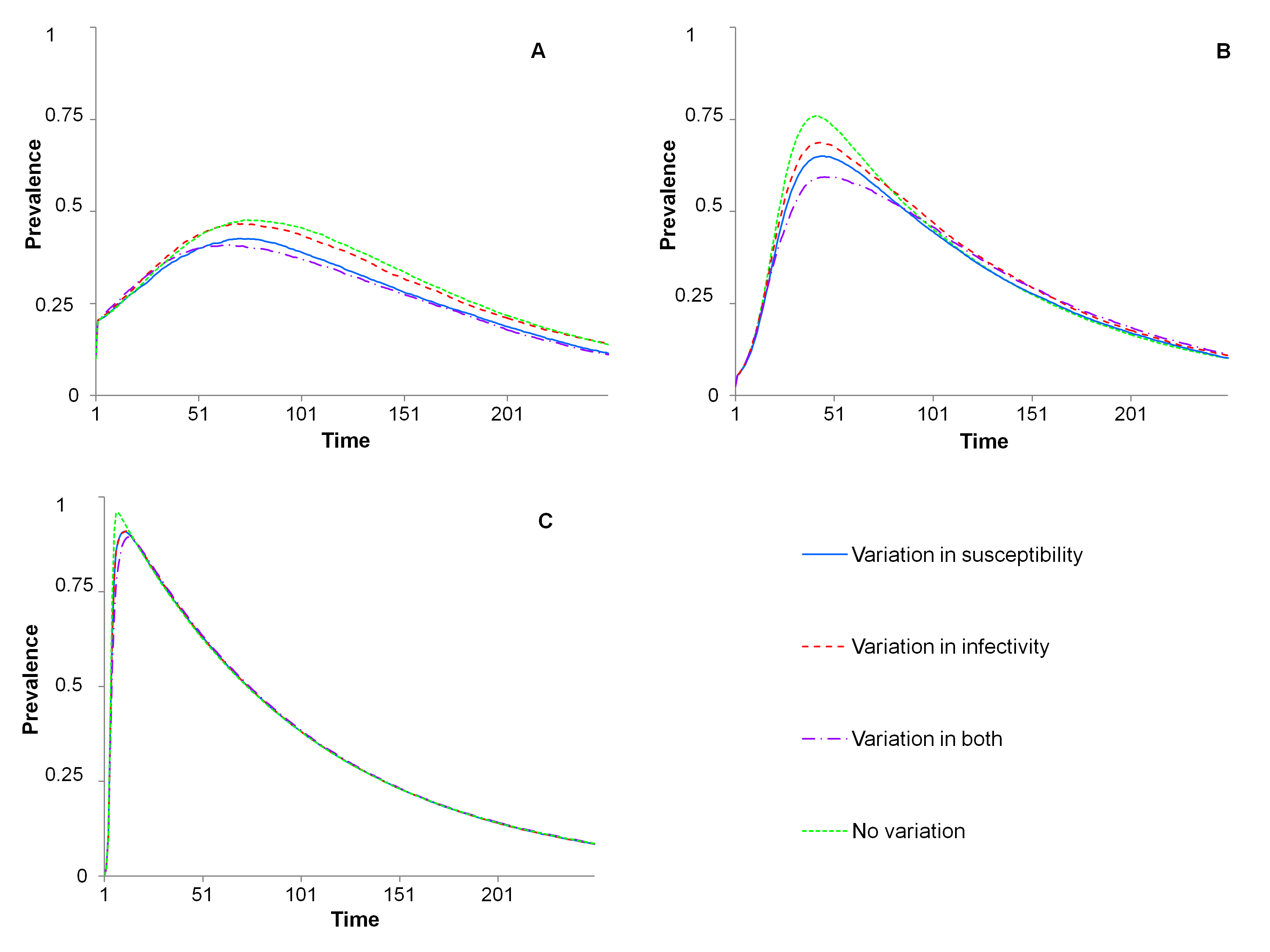

Supplement: Figure S3 — Disease prevalence over time assuming two alleles code for susceptibility or infectivity and a symmetrical distribution. Parameters as in Table 2. Population structure parameters as in Table S1. A) groupsize of 10 B) groupsize of 40 C) groupsize of 400. (TIF) [file pone.0039551.s003.tif]
