# Supplementary material for: Indirect Genetic Effects and the Spread of Infectious Disease: Are We Capturing the Full Heritable Variation Underlying Disease Prevalence?
Source: PLoS One. 2012 Jun 29;7(6):e39551. doi: 10.1371/journal.pone.0039551 (PMC3387195; doi:10.1371/journal.pone.0039551)
Supplement: Table S1 — Population structure parameters. (DOCX) [file pone.0039551.s004.docx]

**Table S1. Population structure parameters**

| Groupsize n | 10 | 40 | 400 |
| --- | --- | --- | --- |
| # Sires *s* | 125 | 500 | 5000 |
| Family size | 40 | 40 | 40 |
| Population size *N* | 5 000 | 20 000 | 200 000 |
| # Groups | 500 | 500 | 500 |
