# Supplementary material for: Indirect Genetic Effects and the Spread of Infectious Disease: Are We Capturing the Full Heritable Variation Underlying Disease Prevalence?
Source: PLoS One. 2012 Jun 29;7(6):e39551. doi: 10.1371/journal.pone.0039551 (PMC3387195; doi:10.1371/journal.pone.0039551)
Supplement: Table S2 — Variance estimates using a logistic link function. (DOCX) [file pone.0039551.s005.docx]

**Table S2. Variance estimates using a logistic link function**

| **Model** | **Conventional:**$\sigma_{D}^{2}$ | **IGE**: | **IGE**: | **IGE**: |
| --- | --- | --- | --- | --- |
|  |  | Direct | Indirect | Direct-Indirect |
| Component | $\sigma_{A}^{2}$ | $\sigma_{D}^{2}$ | $\sigma_{S}^{2}$ | $\sigma_{DS}$ |
| Estimate | 0.178±0.004 | 0.210±0.005 | 0.009±0.001 | 0.024±0.002 |

Estimates averaged over ten replicates. Parameters as in Table 2, 10000 groups of size 10. Values ± standard error.
