# Supplementary material for: Indirect Genetic Effects and the Spread of Infectious Disease: Are We Capturing the Full Heritable Variation Underlying Disease Prevalence?
Source: PLoS One. 2012 Jun 29;7(6):e39551. doi: 10.1371/journal.pone.0039551 (PMC3387195; doi:10.1371/journal.pone.0039551)
Supplement: Table S3 — Mean susceptibility and infectivity following selection using the conventional animal model or the Indirect Genetic Effects model with a logistic link function. (DOCX) [file pone.0039551.s006.docx]

**Table S3. Mean susceptibility and infectivity following selection using the conventional animal model or the Indirect Genetic Effects model with a logistic link function**.

| **Selection** | | | Mean susceptibility | Mean infectivity | R_0_ |
| --- | --- | --- | --- | --- | --- |
| **None** | | | 0.22 | 0.22 | 4.46 |
| **Conventional animal effect** | | EBV | 0.10 | 0.22 | 2.14±0.04 |
| **Direct effect** | | EBV_D_ | 0.11 | 0.21 | 2.06±0.03 |
| **Indirect effect** | | EBV_s_ | 0.13 | 0.18 | 2.13±0.06 |
| **Index** | Ix=EBV_D_+$\bar{p}$ (n-1) EBV_s_ | | 0.11 | 0.20 | 2.03±0.03 |

Population with variation in both infectivity and susceptibility following a skewed multiple allele genetic architecture. 10000 groups of size 10. Proportion selected was 0.10. Values ± standard error when greater than 0.005.
