# Supplementary material for: Indirect Genetic Effects and the Spread of Infectious Disease: Are We Capturing the Full Heritable Variation Underlying Disease Prevalence?
Source: PLoS One. 2012 Jun 29;7(6):e39551. doi: 10.1371/journal.pone.0039551 (PMC3387195; doi:10.1371/journal.pone.0039551)
Supplement: Text S1 — Derivation of transmission parameter from first principles. (DOC) [file pone.0039551.s007.doc]

**Text S1 Derivation of transmission parameter from first principles.**

We define the probability of a susceptible individual *j* to become infected upon contact with an infected individual k as the product of the susceptibility of *j* (g_j_) with the infectivity of *k* (f_k_), with the assumption that susceptibility and infectivity are independent. Let *c_jk_* be the expected number of contacts between individuals *j* and *k* per time unit. The probability of a susceptible individual *j* to avoid getting infected per time unit will therefore be equal to

$$\prod_{k=1}^{n-1} (1-g_{j}X_{f,k}f_{k})^{c_{jk}}.$$

The indicator trait *X_f,k_* is equal to one if *k* is infected and zero otherwise.

The probability *dq* of individual *j* to become infected during a time period *dt* is therefore:

$$\begin{matrix} dq & =1-\left(\prod_{k=1}^{n-1} (1-g_{j}X_{f,k}f_{k})^{c_{jk}}\right)^{dt} \\ \text{ } & =1-\exp\left(dt\sum_{k=1}^{n-1} c_{jk}\ln(1-g_{j}X_{f,k}f_{k})\right).\text{ } \end{matrix}$$

Using the property, $e^{x}=1+x+x^{2}/2!+x^{3}/3!+...$, dividing by *dt* and taking *lim dt→0*, we obtain the rate of infection for one individual or force of infection,

$$\frac{dq}{dt}=-\sum_{k=1}^{n-1} c_{jk}ln(1-g_{j}X_{f,k}f_{k}).$$

Hence, the change in the number of susceptible individuals over a time period dt is given as:

$$\frac{dS}{dt}=\sum_{j=1}^{n} \sum_{k=1}^{n-1} c_{jk}ln(1-X_{g,j}g_{j}X_{f,k}f_{k}).$$

The indicator trait *X_g,j_* is equal to one if *j* is susceptible and zero otherwise.

The pairwise transmission parameter *β_jk_* is defined as the rate at which a susceptible individual j will become infected upon contact with an infected individual k. In this way,

$$\beta_{jk}=-ln(1-X_{g,j}g_{j}X_{f,k}f_{k}).$$

Note that for small values of *g* and *f* this may be approximated by $\beta_{jk}=X_{g,j}g_{j}X_{f,k}f_{k}$.
