# Supplementary material for: Indirect Genetic Effects and the Spread of Infectious Disease: Are We Capturing the Full Heritable Variation Underlying Disease Prevalence?
Source: PLoS One. 2012 Jun 29;7(6):e39551. doi: 10.1371/journal.pone.0039551 (PMC3387195; doi:10.1371/journal.pone.0039551)
Supplement: Text S2 — Derivation of variance in disease presence. (DOC) [file pone.0039551.s008.doc]

**Text S2 Derivation of variance in disease presence.**

Assuming that disease presence is distributed according to equation (4) and that the environmental component is independent from all other components, the variance in disease presence can be expressed as follows:

$$\begin{matrix} \sigma^{2} & =\text{cov}(b_{1}g+b_{2}\sum_{m=1}^{p} f_{m}+e,b_{1}g+b_{2}\sum_{m=1}^{p} f_{m}+e) \\ \text{ } & =b_{1}^{2}\sigma_{g}^{2}+2b_{1}b_{2}\text{cov}(g,\sum_{m=1}^{p} f_{m})+b_{2}^{2}\text{var}(\sum_{m=1}^{p} f_{m})+\sigma_{e}^{2} \\ & \end{matrix}$$

Assuming that the number of individuals which have been infected *p* is a random variable and given independence between input susceptibility and infectivity,

$$\begin{matrix} & \text{cov}(g,\sum_{m=1}^{p} f_{m}) & = & \text{E}_{p}\left(\text{cov}(g,\sum_{m=1}^{p} f_{m}|p)\right)+\text{cov}_{p}\left(\text{E}(g),\text{E}(\sum_{m=1}^{p} f_{m}|p)\right) \\ \text{ } & & = & \text{E}(p)\text{cov}(g,f)+0 \\ \text{ } & & = & 0 \\ \text{ } & & & \end{matrix}$$

$$\begin{matrix} & \text{var}(\sum_{m=1}^{p} f_{m}) & = & \text{E}_{p}\left(\text{var}(\sum_{m=1}^{p} f_{m}|p)\right)+\text{var}_{p}\left(\text{E}(\sum_{m=1}^{p} f_{m}|p)\right) \\ & & = & \text{E}(p)\text{var}(f)+\text{E}^{2}(f)\text{var}(p) \\ & & = & \bar{p}\sigma_{f}^{2}+\bar{f}^{2}\sigma_{p}^{2}. \\ & & & \end{matrix}$$

Incorporating equations (14) and (15) into equation (13) we obtain equation (5).
