# Supplementary material for: Indirect Genetic Effects and the Spread of Infectious Disease: Are We Capturing the Full Heritable Variation Underlying Disease Prevalence?
Source: PLoS One. 2012 Jun 29;7(6):e39551. doi: 10.1371/journal.pone.0039551 (PMC3387195; doi:10.1371/journal.pone.0039551)
Supplement: Text S3 — Impact of model parameters on prevalence profiles. (DOC) [file pone.0039551.s009.doc]

**Text S3 Impact of model parameters on prevalence profiles**

The desired outcome of control of infectious diseases through selection is a reduction in prevalence. Moreover, genetic parameter estimates of disease traits depend on disease prevalence (Bishop and Woolliams 2010). The impact of the genetic model parameters on prevalence profile was therefore examined.

**Impact of mean susceptibility/infectivity on prevalence profiles:** In order to examine the impact of different levels of infectivity or susceptibility on disease prevalence in our model we first ran simulations for homogeneous populations with two levels of infectivity *f* and susceptibility *g*. Specifically, for both infectivity and susceptibility the high level equals 0.4 and the low level 0.04. From Figure S1 it is clear that populations with different degrees of susceptibility/infectivity have different prevalence profiles. Note that, populations with a high level of susceptibility and low infectivity had the same expected prevalence over time as populations with low susceptibility and high infectivity (cf. Figure S1). In other words, different levels of infectivity or susceptibility have the same impact on disease prevalence in this model.

**Impact of variation in susceptibility and/or infectivity:** The impact of variation in susceptibility and/or infectivity on disease prevalence at different stages of the epidemic for the different genetic models (i.e. bi-allelic vs multiple alleles; symmetric vs skewed) and different group sizes was examined. Figures S2 & S3 show the prevalence profile for populations, consisting of different group sizes, with variation introduced in either susceptibility or infectivity, neither or both traits using the skewed multiple allele and symmetric bi-allelic models respectively. Underlying genetic architecture and frequency distribution, however, had little impact on the time course of the epidemic (cf. Figure S2 & S3). Groupsize had the highest impact as with increasing groupsize the epidemic progressed faster towards its maximum prevalence and this maximum prevalence was increased (cf. Figure S2 & S3). The introduction of variation in susceptibility/infectivity had little impact on prevalence profiles although it slightly decreased disease peak prevalence. For all group sizes, the impact of heterogeneity was strongest when there was variation in both susceptibility and infectivity.
