# Supplementary material for: Indirect Genetic Effects and the Spread of Infectious Disease: Are We Capturing the Full Heritable Variation Underlying Disease Prevalence?
Source: PLoS One. 2012 Jun 29;7(6):e39551. doi: 10.1371/journal.pone.0039551 (PMC3387195; doi:10.1371/journal.pone.0039551)
Supplement: Text S4 — Impact of a logistic regression on variance estimates and selection response. (DOC) [file pone.0039551.s010.doc]

**Text S4 Impact of a logistic regression on variance estimates and selection response**

Generalized linear mixed models (GLMM), linking a linear mixed model with a non-linear link function, such as a logistic regression, are often used for the genetic analysis of binary data . It is therefore of interest to assess whether the use of a non-linear link function would alter the main messages of this paper.

For this purpose, the data from the population with variation in susceptibility and infectivity following a skewed multiple allele distribution was analysed with the conventional and the indirect genetic effect (IGE) model (equations 2 and 3) using a logistic link function. The variance estimates obtained from these analyses are displayed in Table S2. Similarly to the linear model without a link function, the animals with the lowest ten percent of estimated breeding values (EBVs) obtained from these analyses were then selected. The mean true values of infectivity, susceptibility and basic reproduction number R_0_ for each selected subpopulation are displayed in Table S3.

Similarly to the results obtained without a link function, analysis with the IGE model with a logistic link function obtains a variance estimate for the direct effect which is approximately of the same magnitude as that obtained with the conventional model as well as a smaller yet significant variance for the indirect effect (see Table S2).

Again, similarly to the results obtained without a link function, selecting on conventional or direct EBVs results in a reduction in mean susceptibility only whereas selecting on indirect EBVs reduces both mean susceptibility and infectivity (see Table S3). The greatest reduction in R_0_ is also obtained by selecting with an index of direct and indirect EBVs (see Table S3).

It must be pointed out that the variance estimates in Table S2 are clearly not on the same scale as for the parameters underlying the data hence a logistic link function is not an appropriate transformation (see Table 2). Moreover, the direct-indirect covariance estimate is much larger than the indirect variance estimate and selection on the indirect EBVs obtained with a logistic link function results in an even greater reduction in mean susceptibility and less reduction in mean infectivity than its linear equivalent (see Tables S2 and 7). This would indicate that the use of a logistic link function perhaps aggravates the bias created by the interaction between expression of infectivity and susceptibility. Besides, selection on all except the indirect EBVs results in a greater reduction in R_0_ when analysing without a logistic link function.
